# Detection of Bacterial Pathogens and Antibiotic Residues in Chicken Meat: A Review

**DOI:** 10.3390/foods9101504

**Published:** 2020-10-20

**Authors:** Harsh Kumar, Kanchan Bhardwaj, Talwinder Kaur, Eugenie Nepovimova, Kamil Kuča, Vinod Kumar, Shashi Kant Bhatia, Daljeet Singh Dhanjal, Chirag Chopra, Reena Singh, Shivani Guleria, Tek Chand Bhalla, Rachna Verma, Dinesh Kumar

**Affiliations:** 1 School of Bioengineering & Food Technology, Shoolini University of Biotechnology and ManagementSciences, Solan 173229, India; microharshs@gmail.com; 2 School of Biological and Environmental Sciences, Shoolini University of Biotechnology and ManagementSciences, Solan 173229, India; kanchankannu1992@gmail.com (K.B.); rachnaverma@shooliniuniversity.com (R.V.); 3 Department of Agriculture, Sri Guru Teg Bahadur Khalsa College, Sri Anandpur Sahib, Punjab 140117, India; talwinderkaur83@gmail.com; 4 Department of Chemistry, Faculty of Science, University of Hradec Kralove,50003 Hradec Kralove, Czech Republic; eugenie.nepovimova@uhk.cz; 5 School of Water, Energy and Environment, Cranfield University, Cranfield MK43 0AL, UK; Vinod.Kumar@cranfield.ac.uk; 6 Department of Biological Engineering, College of Engineering, Konkuk University, Seoul 05029, Korea; shashikonkukuni@konkuk.ac.kr; 7 School of Bioengineering and Biosciences, Lovely Professional University, Phagwara, Punjab 144411, India; daljeetdhanjal92@gmail.com (D.S.D.); chirag.18298@lpu.co.in (C.C.); reena.19408@lpu.co.in (R.S.); 8 Department of Biotechnology, TIFAC-Centre of Relevance and Excellence in Agro and Industrial Biotechnology (CORE), Thapar Institute of Engineering and Technology, Patiala 147001, India; shivani.guleria55@gmail.com; 9 Department of Biotechnology, Himachal Pradesh University, Summer Hill, Shimla 171005, India; bhallatc@rediffmail.com

**Keywords:** antibiotics, multidrug-resistant bacteria, biosensors, chicken, chromatography-based method, molecular-based methods, immunology-based method

## Abstract

Detection of pathogenic microbes as well as antibiotic residues in food animals, especially in chicken, has become a matter of food security worldwide. The association of various pathogenic bacteria in different diseases and selective pressure induced by accumulated antibiotic residue to develop antibiotic resistance is also emerging as the threat to human health. These challenges have made the containment of pathogenic bacteria and early detection of antibiotic residue highly crucial for robust and precise detection. However, the traditional culture-based approaches are well-comprehended for identifying microbes. Nevertheless, because they are inadequate, time-consuming and laborious, these conventional methods are not predominantly used. Therefore, it has become essential to explore alternatives for the easy and robust detection of pathogenic microbes and antibiotic residue in the food source. Presently, different monitoring, as well as detection techniques like PCR-based, assay (nucleic acid)-based, enzyme-linked immunosorbent assays (ELISA)-based, aptamer-based, biosensor-based, matrix-assisted laser desorption/ionization-time of flight mass spectrometry-based and electronic nose-based methods, have been developed for detecting the presence of bacterial contaminants and antibiotic residues. The current review intends to summarize the different techniques and underline the potential of every method used for the detection of bacterial pathogens and antibiotic residue in chicken meat.

## 1. Introduction

Globally, both developed and developing countries predominantly consume chicken as a meat product. According to the report of Global Livestock Counts, there are around 19 billion chickens in the world [1]. In 2019, consumption of chicken meat in the USA was 16,700 metric tons; in the European Union it was 11,636 metric tons, and in India, it was 4347 metric tons [2]. Chicken meat is stated as white meat, which makes it distinct from other meats like lamb and beef, owing to its low iron content and its lack oftrans-fat. Moreover, no trans-fats make it a healthier option as they are associated with cardiovascular disease, whereas beef and lamb meat contains a high amount of trans-fat [3].

Over the past few decades, different countries have undergone substantial changes in eating habits. With changing lifestyles, people now frequently go out for meals, and middle-class people most often consume chicken meat. Additionally, the relocation of people from rural to urban areas has also contributed to the change in eating patterns [4]. Innovative distribution, preparation and food production techniques have also been found to be responsible for these changes. If necessary preventive measures are not taken into account during marketing, processing and production of chicken, there are chances that chicken eggs and meat can get contaminated via infectious agents which could be pathogenic to humans [4]. *Campylobacter* and *Salmonella* are the common pathogenic microbes accounting for >90% cases of food poisonings associated with bacteria and are considered responsible for food safety hazards worldwide [4]. The list of outbreaks associated with chicken/meat consumption has been compiled in Table 1.

*Escherichia coli* and *Salmonella* are the predominant bacteria found in the intestines of both animals and humans. These microbes serve as an indicator of fecal contamination in food and water bodies due to the untreated discharge of municipal wastewater in natural water streams [1]. Chicken with *E. coli* contamination shows the inadequate practice of hygiene in slaughterhouse and trading areas [1]. As per the report of the European Food Safety Authority (EFSA), *Campylobacter* spp. is a leading food-borne hazard associated with poultry meat, due to cross-contamination during processing in contaminated broiler and the packaging of ready-to-use foods [19]. Moreover, it has been estimated that 50–80% of cases of campylobacteriosis in humans are due to poor handling of the chicken reservoir, whereas, 20–30% can be attributed to consumption of contaminated broiler meat and poor handling during meat preparation [20].

Therefore, to circumvent the problem of contamination, poultry industries have started using antibiotics to enhance the production of meat using enriched feed for disease prevention [21]. The common antibiotics used in chicken farming, along with their biological effects, are shown in Table 2.

Productive application of antibiotics in poultry has substantially improved the growth and health of birds by boosting their immune system [21]. However, the presence of antibiotic residues in meat imposes a problem in humans, as antibiotic residues can elicit an allergic response, imbalance of intestinal microbiota and in few cases, it can lead to the development of resistance against antibiotics [33]. Hence, the purpose of this review is to provide complete knowledge of the conventional and advanced methods available for the detection of bacterial pathogens and antibiotic residue in chicken meat.

## 2. Source of Bacterial Contamination

According to published literature, there are only two ways of inducing infection in eggshell by bacteria, i.e., horizontal or vertical transmission. In vertical transmission, it occurs through the systemic infection of ovaries or during intercourse with a contaminated cloaca, touching the vagina and lower regions of the oviduct [34]. During the vertical transmission, the yolk, albumen and membrane come into direct contact with the contaminants due to bacterial infection of reproductive organs like oviduct tissue and ovaries. As a result, eggs get contaminated before the formation of the shell [35]. *Campylobacter* and *Salmonella* are the bacterial species which predominantly contaminates the eggs via this route of infection. On the other hand, horizontal transmission takes place through broken eggs, blood, hands, insects and water.

Additionally, it also gets transmitted horizontally due to the interaction with dust, feces and soil during transportation and from caging material [34]. The ubiquitous nature of bacteria allows them to contaminate boiler meat [36]. Moreover, the cut meat, as well as the equipment used for cutting, comes into direct contact with air, which easily contaminates the meat. In fresh meat, bacteria are mostly found on the surface instead of inside the meat [37]. On the other hand, in processed meat, as they are marinated, the bacteria get easily penetrated in the muscles [38]. Although water has washing effects, and it decreases the bacterial load during the processing of meat, it also increases the chances of cross-contamination between carcasses [39,40].

## 3. Conventional Methods of Microbial Detection

### 3.1. Culture-Based Method

Back in the 19th century, bacterial culture was first introduced. Before that, many biologists were trying to grow bacteria on food or other material on which microbes were first observed [41]. Culture-based methods are the oldest methods used for detecting microbes, even pathogenic strains, as their result confirms the presence of a particular microbe [42]. Culture-based methods are a subtle but time-intensive process [43]. Some bacterial species require an enrichment broth or buffer before their isolation on differential media and serological confirmation [44]. Numerous chicken-borne pathogenic bacteria species confirmed via a culture-based method have been enlisted in Table 3.

*Salmonella* spp. isolation from chicken, egg and meat products buffered with peptone water, selenite cystine, tetrathionate (TT) or Rappaport-Vassiliadis (RV) are used as the enrichment broth, and brilliant green agar, hektoen enteric agar and xylose lysine deoxycholate (XLD) agar are used as selective media [57,64,68]. Selective media like blood agar, Eosin Methylene Blue (EMB) Agar or MacConkey are used for isolating *E. coli* [51,60]. Moreover, *Campylobacter* spp. as well as *Enterococcus* spp. are isolated with the help of enterococcosel agar and Preston agar [65,69]. Furthermore, *Staphylococcus* species (both coagulase-positive and negative) are extensively isolated by Baird parker agar and mannitol salt agar [70].

### 3.2. PCR-Based Method

A polymerase chain reaction (PCR) is also a detection approach as it allows us to robustly replicate the desired DNA segment and serve the dual purpose, i.e., detection and identification of particular species [71]. This process uses a specific set of primers for replicating the desired segment of DNA by following three steps: denaturation, annealing and extension. All these steps work under the desired range of temperature, i.e., denaturation: 90–95 °C (high-temperature), annealing: 55–60 °C (low temperature) and extension: 70–72 °C (intermediate temperature) [72]. PCR approaches are sensitive, precise, detect different pathogenic microbes simultaneously and minimize the risk of contamination, but require highly trained personnel and a robust thermal cycler [43]. The chicken-borne bacterial pathogens identified using different types of PCR have been enlisted in Table 4.

Arunrut et al. [82] developed a real-time loop-mediated isothermal amplification (LAMP) procedure for the identification of *Salmonella* spp. with the help gene62181533 as a primer sequence. This procedure did not display any cross-reactivity with other pathogenic bacteria. Moreover, spiked chicken sample results obtained for the accuracy, specificity and sensitivity of this procedure were found to be 90.83%, 86.79% and 94.02%, respectively. Alves et al. developed a multiplex-PCR procedure, using specific primers for *Campylobacter* spp., i.e., OT1559 and 18-1 primers, as well as specific primers for *Salmonella* spp., i.e., Styinva-JHO-Right and Styinva-JHO-Left primers [83]. The specificity of the assay was found to be 100% and it was able to detect 1 cfu/mL of *Salmonella* spp. (after nonselective enrichment) and 10^2^ cfu/mL of *Campylobacter* spp. (after selective enrichment). Another study was conducted for comparative analysis of four PCR kits that are commercially available for the detection of *E. coli*, *E. coli* O157-H7, *Salmonella* spp., and *S. aureus*, in both artificially and naturally contaminated chicken products. The specificity of the kits for *E. coli* O157-H7, *E. coli*, *Salmonella* spp. and *S. aureus* in chicken products were found to be 95%, 97%, 96% and 100%, respectively [84].

### 3.3. Array-Based Method

Array signifies miniature, the two-dimensional high-density matrix of DNA fragments printed over the silicon or glass slide in a distinctive manner. This chip is used for the hybridization of DNA fragments to fluorescent-labelled probes for detection via advanced instrumentation and software [85]. For instance, Microbial Diagnostic Microarrays (MDMs) employs three different kinds of probes like short oligonucleotides, long oligonucleotides and PCR amplicons. Short oligonucleotides show a low binding affinity due to mismatch in 1–2 nucleotide; hence amplification with PCR becomes evident [85]. On the other hand, long oligonucleotides and PCR amplicons show a higher binding affinity and a lower discrimination potential. Therefore, both long oligonucleotides and PCR amplicons can be used in combination with generic amplification approaches like whole genome amplification (WGA). This method is array-based and can robustly identify pathogens. However, this technique is expensive and needs skilled personnel [43]. A list of chicken-borne bacterial pathogens assessed by different types of arrays is shown in Table 5.

Quiñones et al. [89] developed a DNA oligonucleotide array for simultaneous detection of *Arcobacter* and *Campylobacter* in retail chicken samples. The probes selected for developing this array were having high affinity for both housekeeping and virulence-associated genes in *Arcobacter butzleri*, *Campylobacter coli* and *Campylobacter jejuni*. Another group of researchers developed a DNA-based bead array for simultaneous determination of 11 pathogens viz. *Escherichia coli*, *E. coli* O157: H7, *Listeria grayi*, *L. ivanovii*, *L. innocua*, *L. welshimeri*, *L. monocytogenes*, *L. seeligeri*, *Salmonella* spp., *S. aureus* and methicillin-resistant *S. aureus* [90]. Apart from this, the bead array method has been developed based on fluorescent-labelled paramagnetic beads attached to unique stretches of 24 oligonucleotide (anti-TAG) sequences. These unique 24 oligonucleotide sequences further bind with a biotinylated PCR product containing a complementary TAG sequence. In this method, R-phycoerythrin labelled streptavidin was utilized to detect the presence of biotinylated PCR products. This method exhibited a relative sensitivity, relative accuracy and relative specificity of 95%, 96% and 100%, respectively.

### 3.4. ELISA-Based Method

One of the most reliable immunoassays used todate is the enzyme-linked immunosorbent assay (ELISA). In this approach, purity of antibody plays a vital role in the specificity, sensitivity and accuracy of this approach [42]. Polyclonal antibodies are not preferred in this approach as multiple epitopes affects specificity as well as the sensitivity of the reaction. The application of different substrates in ELISA has an additional advantage because specific substrates bind with respective conjugate and produce coloration, which could either be read through an ELISA reader in wavelength and color change can be visualized with the naked eye [42]. ELISA can precisely detect the microbial contaminants as well as their toxins and ELISA kits have been developed for identifying pathogenic microbes and toxins according to the requirement [43]. Various ELISA-based methods developed for the detection of chicken-borne pathogenic bacteria have been compiled in Table 6.

Schneid et al. [91] developed an indirect ELISA approach to detect *Salmonella enterica serovar Enteritidis* in a sample of chicken. For this, to improve the sensitivity, four wells of polystyrene plates were filled with wholly killed cells of bacteria along with monoclonal antibodies and were incubated at 37 °C for 1 h. After incubation, the prepared sample was washed with protein A-peroxide conjugated antibodies, and again the sample was incubated at 37 °C for 1 h, to observe the result by adding chromogenic substrate to the treated sample. Out of 154 tested samples, approximately 26% showed the positive result for the presence of bacteria via ELISA. Lilja and Hänninen [93] conducted another study to assess the quality of a commercially available ELISA kit to identify *Campylobacter* spp. contamination in retail meat samples of chicken. Out of 97 tested samples, only 13 showed the positive result for *Campylobacter* by ELISA. Vanderlinde and Grau [95] conducted a similar study, but they used the ELISA kit for the detection of Listeria spp. in the retail meat sample, for which 72 samples showed a positive result for the presence of *Listeria* spp. out of 74 samples.

Charlermroj et al. [96] conducted the study by using an immune-bead array approach for the simultaneous detection of three food-borne pathogens, i.e., *Campylobacter jejuni*, *Salmonella* spp. and *Listeria monocytogenes*. This array method used the sandwich ELISA principle for the detection of these pathogenic bacteria. In this study, three sets of fluorescently labelled beads were used. Each labelled bead was attached to capturing antibodies specific to pathogenic bacteria, whereas detecting antibodies were labelled with R-phycoerythrin (RPE) having the affinity and specificity for capturing antibodies. This process allows the detector to detect the signals of both labelled beads as well as that of the RPE molecule attached to the detecting antibody. This method was used for assessing the presence of pathogenic bacteria in both ready-to-cook (RTC) as well as ready-to-eat (RTE) chicken products. Moreover, this method was found to be effective in detecting spiked pathogenic bacteria at 1cfu in both types of food sample [96].

## 4. Advanced Methods of Detection

### 4.1. Aptamers Based Method

Aptamers are short stretches of single-stranded biomolecules (ssRNA or ssDNA) of 15–80 nucleotide length. These form a three-dimensional structure, which can interact with targeted molecules via base stacking, electrostatic interactions, Van der Waals forces, hydrogen bonding or a combination of these interactions [97]. Synthetic ssRNA or ssDNA libraries are evaluated for identifying aptamers via a process named “Systematic Evolution of Ligands by EXponential enrichment (SELEX)” [98]. Moreover, these aptamers can be developed according to targets ranging from whole cells to ions. Although numerous, aptasensors have been developed for detecting various bacterial pathogens [99,100,101]. Aptamers work in diverse ecological conditions, have a long shelf life and are applicable to a variety of targets. However, RNA-based aptamers have a drawback. RNA-based aptamers degrade very rapidly due to the presence of nucleases in biological media, and in blood in particular, which imposes as a serious issue. Moreover, there are cross-reactivity issues with these aptamers, which restrict the practical application of this approach [102,103]. Aptamers developed for detecting chicken-borne bacterial pathogens have been listed in Table 7.

Renuka et al. [108] developed fluorescent dual aptasensors for onsite sensitive and robust detection of *E. coli* O157: H7 and assessed its authenticity on different food matrices. In this ssDNA, aptamers labelled with biotin were immobilized on silane-glutaraldehyde functionalized glass slides, which act as capturing ligands and aptamers labelled with quantum dots (QDs) act as revealing probes. The method did not show any cross-reactivity with other pathogenic bacteria. Moreover, a spiked meat sample of chicken showed arecovery rate of 76%. Another group of researchers developed an aptamer linked immunosorbent assay (ALISA) for the detection of enterotoxin B synthesized by *Staphylococcus* sp. in ready-to-eat (RTE) chicken [109]. This aptamer-based method was found to be cost-effective, thermally stable and sensitive in contrast to PCR assays. Additionally, aptamers can be developed for the molecules which do not have available antibodies.

Feng et al. [110] reported the precise and efficient system based on loop-mediated isothermal amplification (AMC-LAMP) and magnetic capture aptamers for the detection of *L. monocytogenes* in the raw chicken sample. For this, a set of aptamers (four different types of aptamers having high binding affinity for *L. monocytogenes*) conjugated to magnetic beads, was used for entrapping *L. monocytogenes.* After entrapping, the aptamer system is incubated at room temperature for 45 min. Later, the incubated sample was used for direct DNA isolation. After isolation, LAMP assays were carried out at 63 °C for 40 min, and the amplified product was visualized with the help of SYBR Green^®^ I staining. The detection limit of AMC-LAMP was found to be 5 cfu mL^−1^ with an assay time of 3 h.

### 4.2. Biosensor-Based Method

The biosensor is a fabricated device encompassing biological entities like an antibody, nucleic acid, receptor or any other bio-recognizing entities, which interacts with an analyte and elicits a response and this response is transformed to an electrical signal via the transducer [111]. The response generated by a biosensor is precise, robust and free from noise and has a precise detection limit. These biosensors can detect a bacterium cell in a rationally small volume and can distinguish one bacterial species from another, and even in the strain of the same species [112,113]. Biosensors are automated systems which demand minimal operator interaction. Nowadays, inexpensive biosensors are available with a simple, portable and easy-to-use design. However, major challenges linked with these biosensors are sample pretreatments like the enrichment of bacteria [114,115]. Numerous biosensors developed for detecting chicken-borne bacterial pathogens have been shown in Table 8.

Kim et al. [123] developed colourimetric-based aptasensors for rapid on-site detection of *Campylobacter coli* and *Campylobacter jejuni* in meat samples of chicken. For this, the two-stage aptasensing platform was fabricated using gold nanoparticle (AuNPs), as they aid in a color change from red to purple due to the accumulation of AuNPs. Moreover, this device does not require pH optimization or additional time for aptamers to get absorbed on AuNPs. This colourimetric-based aptasensor has a high specificity towards viable cells of *C. coli* and C. *jejuni.* In another study, the electrochemical impedance spectroscopy technique was used to check the presence of *E. coli* K12 in a meat sample of frozen chicken [124]. For this, antibodies synthesized against *E. coli* were immobilized on to the gold surface via a physisorption method. The binding of antibodies against *E. coli* and *E. coli* K12 on the gold surface was determined with the detection limit of 10^3^ cfu mL^−1^. Huang et al. [125] developed an enzyme-free biosensor for precise and targeted detection of *Salmonella typhimurium* with the help of curcumin (CUR) and 1,2,4,5-tetrazine (Tz)–trans-cyclooctene (TCO) acting as a signal reporter and a signal amplifier, respectively. For fabricating this biosensor, nanoparticles containing bovine serum albumin (BSA) and CUR were reacted with TCO and Tz to synthesize Tz-TCO-CUR conjugates. This Tz-TCO-CUR conjugate was further conjugated with anti-*S. typhimurium* polyclonal antibodies (pAbs) to develop a CUR-TCO-Tz-pAb conjugate.

Furthermore, monoclonal antibodies (mAbs) specifically against *S. typhimurium* were conjugated with Magnetic nanoparticles (MNPs) via streptavidin-biotin binding for effective and targeted separation of *S. typhimurium.* Then, *CUR-TCO-Tz-pAb* conjugates were reacted with MNP-conjugate. The conjugation of both conjugates in the presence of NaOH led to the color change, and color change was used for the determination of S. *typhimurium* contamination. The detection limit of this biosensor was found to be 50 cfu mL^−1^ in the meat sample of chicken spiked with *S. typhimurium*.

### 4.3. Matrix-Assisted Laser Desorption/Ionization-Time of Flight Mass Spectrometry-Based Method

Todate, MALDI-TOF MS is the most predominantly used method to analyze the biomolecules [126]. It works on the principle of ionization of the co-crystallized sample via short laser pulses. As a result, ion gets accelerated, and the time taken by biomolecules to reach the detector is measured. This approach is useful for determining the mass of peptide and protein, along with the mass of unknown protein [127]. Now, it has also been used for differentiating various bacterial species [126]. On the other hand, MALDI-TOF MS can provide an analysis of the peptide fingerprints of microbial proteins that are well-conserved within a species, which enables the characterization of these proteins and their correlation with different species [128]. However, there are a few exceptions, where this method is unable to discriminate between related species because of the inherent similarity of the organisms. For instance, MALDI-TOF MS is incapable of differentiating *Shigella* from *E. coli*. This could likely be because these may not be two species, but one, as it has been stated by taxonomists [128]. Another reason for incorrect identification of similar species could be due to a lack of a consolidated knowledgebase.

Rasmussen et al. [129] used MALDI-TOF MS to examine the presence of β-lactamases synthesized by *E. coli* in both local and imported chicken meat sold in the Ghana market. The result obtained revealed that 153 out of 188 samples contained *E. coli*, and out of this 29 *E. coli* showed the presence of β-lactamase. A similar study was conducted in Egypt on chicken meat bought from the retail shop to assess the presence of β-lactamase/carbapenemase-synthesizing *Enterobacteriaceae* species with the help of MALDI-TOF MS [130]. The result obtained revealed that 69 isolates out of 106 were a β-lactamase producer. In Thailand, MALDI-TOF MS was used to identify the bacterial contaminants present in chicken meat sold in the open meat market. The result obtained showed the presence of 11 different bacterial species viz. *Aeromonas caviae*, *A. veronii*, *Citrobacter freundii*, *Enterobacter asburiae*, *E. coli*, *Klebsiella pneumoniae*, *Lactococcus lactis*, *Staphylococcus warneri*, *S. epidermidis*, *S. pasteuri* and *Serratia fonticola* in chicken breast [131]. In Poland, the evaluation of chicken with MALDI-TOF MS revealed the presence of ciprofloxacin as well as tetracycline-resistant *C. coli* and *C. jejuni* [132].

### 4.4. Electronic Nose-Based Method

The term “Electronic Nose” is an array of chemical gas sensors with a broad spectrum of selectivity to measure the volatile mixture contained inside the headspace over the sample to interpret the presence of specific chemical gas via computer-assisted statistic processing tools [133]. In its working mechanism, the prime neurons, i.e., the chemical sensor of the electronic nose has a precise sensitivity towards different odors. The interaction among the gas sensor and odor compounds elicit the change in sensors, which generates an electrical impulse. The generated electrical impulse is further recorded by an analogue instrument with the secondary neurons. In this manner, signals generated by individual sensors form a unique pattern for the gaseous mixture and are deciphered via an artificial neural network (i.e., a multivariate pattern recognition method) [134].

Rajamaki et al. [135] used an electronic nose method to assess the quality of the modified atmosphere (MA)-packaged broiler chicken pieces. The results obtained from this study were also compared with the results of sensory, microbiological and headspace gas chromatography. In this study, the electronic nose method was found to be effective in distinguishing low-quality packed broiler chicken from freshly packed chicken either formerly or on-the-spot as the quality deteriorates. Timsorn et al. [136] modified the e-nose-based method by attaching it with eight metal oxide semiconductor (MOS) sensors to evaluate the freshness of chicken meat and the know population of bacterial contaminants on chicken meat stored at 4 °C as well as 30 °C for up to five days. The result obtained from this study showed a positive correlation (0.94) with the bacterial population on chicken, signifying that the e-nose method is an effective and robust approach to assess the bacterial population in chicken meat with a high accuracy.

## 5. Conventional Methods of Antibiotics Residue Detection

### 5.1. Microbial Inhibition Test

The microbial inhibition method is predominantly used for assessing the presence of antibiotic residues in food products of animal origin [137]. This method is also time-consuming and laborious like the culture-based method. The test is performed in both plate and test tubes and, in test tubes, the viable culture of bacteria is mixed with a pH or a redox indicator to detect the residues of antibiotics in food samples [138]. In Europe, a microbial inhibition test was conducted in the plate to check the presence of antibiotic residue in slaughtered animals [139,140]. Moreover, a 3-plate test cultured with three different bacterial species like *Bacillus subtilis*, *Escherichia coli* and *Staphylococcus aureus* was used to reveal the presence of antibiotic residues in the organs of poultry animals [141]. The European Union introduced four plate tests for detecting antibiotic residue in meat, in which one plate has a culture of *Micrococcus luteus*, and other three plates have a culture of *Bacillus subtilis* (Table 9) [142].

### 5.2. ELISA-Based Method

Shahbazi et al. [145] conducted an experiment by using the ELISA method to determine the level of tetracycline in a meat sample and reported the mean value to be 247.32 μg kg^−1^. Ramatla et al. [150] conducted a similar study, but they assessed the streptomycin residue level. The mean residue value determined for this study was found to be 647.09 μg kg^−1^, which was higher than the international maximum residue limits (MRL), i.e., 600 μg kg^−1^. Moreover, the concentration of sulphonamide was determined to be 61.01 μg kg^−1^ in the different organs of animals, which is below the recommended MRL, i.e., 100 μg kg^−1^. Additionally, the concentration of tetracycline was also determined and was found to be 168.02 μg kg^−1^. In another study, the residue level of chloramphenicol, streptomycin, sulfamethazine and tetracycline were determined and were found to be in the range of 74 ppb kg^−1^, 30–55 ppb kg^−1^, 1.07–5.60 ppb kg^−1^ and 35–56 ppb kg^−1^, respectively. These values were lower than the acceptable limit, i.e., 100 ppb kg^−1^ established by the EU’s law of drugs [151].

Zhang et al. [152] determined the spiked chloramphenicol recovery rate in chicken muscles in the range of 97–118% via chemiluminescent-ELISA. Various ELISA-based methods used to determine the residues of the antibiotics in chicken meats are shown in Table 10.

### 5.3. Thin-Layer Chromatography (TLC)-Based Method

TLC is the most predominantly used laboratory method worldwide for food as well as quality control analysis [157]. Different adsorption, ion-exchange and partition layers are used for analyzing the food material, but most of the separations are carried out on a stationary phase with pre-coated silica gel. Alumina and cellulose are used as a stationary phase for some food samples. It is a one-dimensional process in which samples ascend from the gravity flow with the help of the mobile phase in the glass chamber. In the detection of analytes, specific chromogenic or fluorogenic reagents are used, and fluorescence detection is highly recommended due to its high sensitivity and specificity. For instance, a high performance-TLC (HPTLC) method was used to examine the presence of nitroimidazoles [157]. The major advantage of TLC is that it is more time-efficient compared with traditional paper chromatography. Minimal equipment is required for the execution of the TLC procedure. For instance, it requires only a fume cupboard, a TLC plate and a TLC chamber. A chamber is an essential component to run a sample that is to be separated into its components. It is effective even if the sample is scarce. Despite its simplicity and convenience, it has a limitation that it cannot differentiate between the enantiomeric and isomeric forms of a compound. Another challenge with TLC is its requirement for pre-known R_f_ values [158]. Different TLC-based methods used for detecting antibiotics residue in chicken meats have been compiled in Table 11.

### 5.4. High-Performance Liquid Chromatography (HPLC)-Based Method

During the 1990s, HPLC gained significant attention as a screening technique due to its automated mode of operation [165]. This approach works on the same principle as chromatography and the detector can be changed according to the nature of the sample under evaluation, as the selection of the detection system is essential for its sensitivity and selectivity. Few samples are not detected via absorbance, and in this case, chromophore, UV-absorbing or fluorescent compounds are used for amending its refractive index or fluorescence, making it suitable for detection [166]. The major advantage of HPLC is its ability to detect multiple residues simultaneously in the sample in a short period. Moreover, developments of high-speed HPLC are highly efficient and require less time for analysis.

Additionally, this system is computer-controlled and fully automated, which makes it an advanced screening technique [165]. HPLC is a costly approach; it requires expensive reagents, columns, a power supply and regular maintenance [167]. Different HPLC-based detection studies conducted to detect the presence of antibiotic residue in chicken meat have been shown in Table 12.

Shalaby et al. [169] used the HPLC approach to assess the presence of tetracycline residue in spiked chicken meat as well as in liver using methanol, acetonitrile and 0.03 M oxalic acid (0:8:92) as the mobile phase. This study revealed that the citrate buffer was more effective in comparison with McIlvaine’s buffer used for matrix extraction. The recovery rate of tetracycline was found to be in the range of 68.7–82.2%. Another study was conducted in which the recovery rate of ten different quinolones (ciprofloxacin, danofloxacin, enrofloxacin, difloxacin, flumequine, lomefloxacin, marbofloxacin, norfloxacin, oxolinic acid, sarafloxacin) were assessed and was found to be 72% in muscle spiked with ten quinolones [169]. In another HPLC-based study, acetonitrile was stated to be an elite extraction solvent for recovering the antibiotic residues of ampicillin, amoxicillin and amoxicillin metabolites from the tissue samples of chicken [172].

## 6. Advanced Methods of Detection

### Biosensor-Based Method

Virolainen et al. [173] and Pikkemaat et al. [174] have published literature about the development of luminescent-based bacterial biosensors for detecting the tetracycline in meat samples. A list of other biosensors developed for the same purpose has been complied in Table 13.

An *E. coli-based* biosensor, in which plasmid containing the *Photorhabdus luminescence*-derived bacterial luciferase operon was used, was placed in such a way so that tetracycline-responsive elements of transposon Tn*10* could control it [173]. Additionally, this controlled system also contains repressor protein TetR, which has an affinity for the operator sequence in P*_tetA_* and helps in reducing the TC binding, which allows transcription from the promoter. Furthermore, usage of bacterial luciferase operon provides a self-luminescent property without any substrate addition to the strain. This characteristic feature makes these cells the sensor element, as they serve as a reagent in the assay.

Gan et al. [176] developed an innovative electrochemical sensor to determine the presence of tetracycline. In this method, the substantial change shown due to the interaction between iron/zinc cations-exchanged montmorillonite layer and tetracycline was measured. Another study stated about amperometric chloramphenicol (CAP) immunosensor for the detection of CAP developed by immobilizing anti-chloramphenicol acetyltransferase (anti-CAT) antibodies on the surface of cadmium sulfide nanoparticles (CdS) modified-dendrimer, which is further bonded with poly 5, 2′: 5′, 2′′-terthiophene-3′-carboxyl acid (poly-TTCA) (conducting polymer). The selection of CdS nanoparticles, dendrimers and gold nanoparticles and their deposition on the polymer layer is made to improve the sensitivity of the probes of this sensor [178].

## 7. Future Prospect

Lately, molecular-based tests, especially mRNA-based tests, have emerged as powerful tools for the robust detection of pathogenic microbes. A limitation of the mRNA-based tests is the instability of the mRNA, which presents as a pitfall in the assessment of food-borne pathogens. Over the last few decades, the lytic phage-based approaches have been developed for the easy and accurate detection of food-borne pathogens in various matrices. Therefore, the combination of both phage amplification and lysis with enzyme assays, PCR/qPCR or immunoassays could be promising alternatives for the detection of viable pathogenic microbes in food. Even aptamer technology and high-throughput sequencing (HTS) approaches have been developed for detecting pathogenic microbes. HTS is now proclaimed to be a robust sequencing approach to sequence a small stretch of genes. The major advantage of HTS is its large sequence output in terms of its entire genome or the large targeted region over the Sanger sequencing method. Hence, the subsequent advancements in the mRNA-based test, phage amplification and lysis with enzyme assays, PCR/qPCR, immunoassays, aptamer technology and HTS for targeted monitoring of pathogenic microbes from different food samples can uplift the detection procedure to a new level. In the future, new rational biosensing and nanomaterials will also likely be used to achieve the robust detection of pathogenic microbes with great precision ([181,182]).

## 8. Conclusions

In the last few decades, there have been substantial improvements in the techniques used for identifying bacterial pathogens and antibiotic residue in food samples and especially in meat. Even though there are limitations associated with culture and microscopy, they are still the predominantly used detection techniques. Genetic and PCR are an effective non-culturable technique used for presently determining the bacterial pathogen, and on the other hand, MS techniques have emerged as an effective method for identifying microorganisms and detecting antibiotic residues. However, these approaches are limited to assess the pure cultures and are ineffective indeciphering the complex samples. To overcome this, chromatography-based methods like TLC and HPLC have been simplified and have eased the challenge associated with MS techniques. In the future, the progressive development and combination of these techniques and instruments will advance the ability to detect the pathogenic microbes and antibiotic residues.

## Figures and Tables

**Table 1:** Disease outbreaks due to chicken/meat consumptions.

Country	Year	Source	Pathogen	Disease	Confirmed Cases	Reference
Canada	2015–2019	Frozen raw breaded chicken products	*Salmonella enteritidis*	*Salmonellosis*	584	[5]
United Kingdom	2017	Chicken liver dishes	*Campylobacter* spp.	*Campylobacteriosis*	7	[6]
India	2016	Cooked chicken	*Clostridium perfringens* or *Bacillus cereus*	Food poisoning	68	[7]
Zimbabwe	2014	Stewed chicken	*Staphylococcus aureus*	Food poisoning	53	[8]
United States	2013–2014	Chicken dishes	*Salmonella* Heidelberg	*Salmonellosis*	634	[9]
Australia	2012	Chicken liver pâté	*Campylobacter* spp.	*Campylobacteriosis*	15	[10]
United States	2012	Undercooked chicken liver	*Campylobacter jejuni*	*Campylobacteriosis*	6	[11]
United Kingdom	2011	Undercooked chicken liver pâté	*Campylobacter coli, Campylobacter jejuni*	*Campylobacteriosis*	22	[12]
United Kingdom	2011	Chicken liver parfait	*Campylobacter* spp.	*Campylobacteriosis*	3	[13]
Australia	2009	Chicken wraps	*Listeria monocytogenes*	*Listeriosis*	36	[14]
United Kingdom	2009	Chicken liver pâté	*Salmonella typhimurium* DT8, *Campylobacter* spp.	*Campylobacteriosis, Salmonellosis*	14	[15]
United Kingdom	2007	Lemon-and-coriander chicken wraps	Verotoxin-producing *Escherichia coli* O157	Diarrhoea	12	[16]
Australia	2005	Chicken dishes	*Campylobacter* spp.	*Campylobacteriosis*	11	[17]
United Kingdom	1984–1985	Live chicken	*Campylobacter jejuni*	*Campylobacteriosis*	19	[18]

**Table 2:** Common antibiotics used in chicken farming.

Name of Antibiotic Class	Types of Antibiotics	Mode of Administration	Biological Effect	Reference
Tetracyclines	Tetracycline; Oxytetracycline; Doxycycline; Chlortetracycline	Oral and intramuscular	Bacteriostatic activity against a wide array of Gram-positive and-negative bacteria, mycoplasmas, some mycobacteria, as well as several protozoa and filariae	[22]
Macrolides	Tylosin; Tilmicosin	Oral	Antibacterial activity against pathogens such as Gram-positive and-negative bacteria	[23]
Lipopeptides	Polymyxins	Oral	Antibacterial activity against Gram-negative bacteria	[24]
Penicillins	Penicillin	Oral	Growth promoter	[25,26]
Folate Pathway Inhibitors	Trimethoprim	Oral	Treatment of respiratory, gastrointestinal infections	[27]
Quinolones	Enrofloxacin; Ciprofloxacin; Danofloxacin	Oral	Growth promoter and antibacterial activity against pathogens such as Gram-positive and-negative bacteria	[28,29,30]
Aminoglycosides	Neomycin; Streptomycin	Oral	Antibacterial activity against Gram-negative bacteria	[31]
Lincosamides	Lincomycin	Oral and intramuscular	Antibacterial activity against Gram-positive bacteria	[32]

**Table 3:** Isolation and identification of pathogenic bacteria using different selective and deferential media along with their antibiotic resistance pattern.

Source of Isolation	Site of Isolation	Types of Medium	Incubation Temperature/Time	Types of Bacteria	Antibiogram Assay	Antibiotics Resistance	Reference
Egg	Shell surface, yolk, albumin	MacConkey agar, Eosin methylene blue (EMB) agar, Bismuth sulphite agar, *Salmonella Shigella* agar, Xylose lysine deoxycholate agar	37 °C/24–48 h	*Citrobacter* spp., *Enterobacter* spp., *Escherichia* spp., *Klebsiella* spp., *Proteus* spp., *Shigella* spp., *Serratia* spp.	Disk diffusion	Cefixime, amoxicillin, amoxyclave	[45]
	Whole egg content	Xylose Lysine Deoxycholate agar, MacConkey agar	37 °C/24–48 h	*Salmonella typhi*, *Salmonella enteritidis*	Disk diffusion	Co-trimoxazole, nalidixic acid, ampicillin, tetracycline, kanamycin	[46]
	Shell surface, interior	Xylose lysine deoxycholate agar, *Salmonella Shigella* agar	37 °C/24 h	*Salmonella* spp.	Disk diffusion	Tetracyclin, ampicillin, amoxicillin	[47]
	Shell surface	Eosin methylene blue (EMB) agar	37 °C/24 h	*Escherichia coli*	Disk diffusion	Penicillin, ciprofloxacin, rifampicin, kanamycin, streptomycin, cefixime, erythromycin, ampicillin, tetracycline	[48]
	Shell surface	*Salmonella Shigella* agar	37 °C/24 h	*Salmonella typhimurium*, *Salmonella enteritidis*	Disk diffusion	Erythromycin, ampicillin, penicillin, tetracycline	[49]
	Shell surface, yolk, albumin	*Salmonella Shigella* agar, Xylose lysine deoxycholate agar, Bismuth sulphite agar	35–37 °C/24 h	*Salmonella enterica* subsp*. salamae*, *Salmonella enterica* subsp*. indica*, *Salmonella* *paratyphi*-A, *Salmonella bongori*, *Salmonella* *choleraesuis*	Disk diffusion	Amoxicillin, ampicillin	[50]
	Yolk	Blood agar, McConkey agar	37 °C/24 h	*Escherichia coli*	Disk diffusion	Ampicillin	[51]
	Interior content	Xylose lysine deoxycholate agar, Bismuth sulphite agar	37 °C/24 h	*Salmonella enteritidis*	MIC	Ampicillin, nalidixic acid, tetracycline	[52]
	Shell surface, interior	McConkey agar	37 °C/24 h	*Escherichia coli*, *Salmonella* spp., *Campylobacter* spp. and *Listeria* spp. *Enterobacter* spp. *Klebsiella* spp.	Disk diffusion	Streptomycin, tetracycline, kanamycin	[53]
	Shell surface	Eosin methylene blue (EMB) agar	37 °C/24 h	*Escherichia coli*	Disk diffusion	Ampicillin, streptomycin, tetracycline	[54]
	Shell surface, interior	Brilliant green agar, McConkey agar, *Salmonella Shigella* agar	37 °C/24 h	*Salmonella enteritidis*	Disk diffusion	Bacitracin, erythromycin, novobiocin	[55]
	Shell surface, yolk	Blood agar, Mannitol salt agar	37 °C/24–48 h	*Staphylococcus aureus*	Disk diffusion	Erythromycin, tetracycline	[56]
	Shell surface, yolk, albumin	Hektoen enteric agar	37 °C/24 h	*Salmonella typhimurium*	Disk diffusion	Bacitracin, polymyxin-B, colistin	[57]
	Shell surface, yolk	Hektoen enteric agar	37 °C/24 h	*Salmonella typhimurium*	Disk diffusion	Clindamycin, oxacillin, penicillin, vancomycin	[58]
ealthy chicken	Skin, feather, nasal, cloaca	Mannitol salt agar, McConkey agar, Brilliant green agar, Blood agar	ND	*Staphylococcus aureus*, *Escherichia coli*, *Pasteurella* spp., *Salmonella* spp.	Disk diffusion	Tetracycline	[59]
	Cloaca	Eosin methylene blue (EMB) agar	ND	*Escherichia coli*	Disk diffusion	Gentamycin, erythromycin, penicillin, cephalexin, amoxicillin, nalidixic acid	[60]
	Cloaca	Xylose lysine deoxycholate agar, Brilliant green agar	37 °C/24 h	*Salmonella* spp.	Disk diffusion	Kanamycin, sulfamethoxazole-trimethoprim, nalidixic acid, ampicillin, cefoxitin, streptomycin, tetracycline, chloramphenicol	[61]
	Cloaca	Xylose lysine deoxycholate agar	ND	*Salmonella* spp.	Disk diffusion	Tetracycline, chloramphenicol, ampicillin, streptomycin	[62]
	Cloaca	McConkey agar, Eosin methylene blue (EMB) agar	37 °C/18–24 h	*Escherichia coli*	Disk diffusion	Ampicillin, tetracycline, sulfamethoxazole-trimethoprim, nalidixic acid	[63]
Meat	Drumsticks, gizzards, liver	Xylose lysine deoxycholate agar, Brilliant green agar	37 °C/24 h	*Salmonella* spp.	Disk diffusion	Erythromycin, penicillin, amoxicillin	[64]
	Liver, gizzards, hearts	Enterococcosel agar	37 °C/48 h	*Enterococcus faecalis*	Disk diffusion	Oxytetracycline, dihydrostreptomycin	[65]
	Brest	Enterococcosel agar	35 °C/24 h	*Enterococcus faecium*	MIC	Quinupristin-dalfopristin	[66]
	Brest, muscle	McConkey agar supplemented with 5% sheep blood	37 °C/18–24 h	*Escherichia coli*	Disk diffusion	Tetracycline, chloramphenicol, nitrofurantoin	[67]

ND—not defined.

**Table 4:** PCR approaches used for the detection of chicken-borne pathogens.

Type of PCR	Sample Used	Target Site of Bacterial Pathogen	Primers	Probe	Detection Chemistry	Limit of Detection	Reference
Simple	Meat (PND)	Spiked *Salmonella typhimurium:ogdh* gene	Forward 5′-GCCTTCCTGAAACGTGACCTA-3′ and reverse 5′-ACCATCTCTTTCAGCATGGGT3′	NA	NA	10^2^ cfu/mL	[73]
Multiplex	Meat (Breasts, wings, drumsticks, legs)	*Clostridium perfringens:cpa*, *cpb*, *etx*, *iA*, *cpe* and *cpb2* genes	Forward 5′-GCTAATGTTACTGCCGTTGA-3′ and reverse 5′-CCTCTGATACATCGTGTAAG-3′; Forward 5′- GCGAATATGCTGAATCATCTA-3′ and reverse 5′-GCAGGAACATTAGTATATCTTC-3′; Forward 5′-GCGGTGATATCCATCTATTC-3′ and reverse 5′-CCACTTACTTGTCCTACTAAC-3′; Forward 5′-ACTACTCTCAGACAAGACAG-3′ and reverse 5′-CTTTCCTTCTATTACTATACG-3′; Forward 5′-GGAGATGGTTGGATATTAGG-3′ and reverse 5′-GGACCAGCAGTTGTAGATA-3′; Forward 5′-AGATTTTAAATATGATCCTAACC-3′ and reverse 5′-CAATACCCTTCACCAAATACTC-3′	NA	NA	NA	[74]
Multiplex Real-Time	Meat (PND)	*Salmonella* spp.: *invA*; *Escherichia coli* O157*: rfbE*; Listeria *monocytogenes*: *hlyA* gene	Forward 5′-GTTGAGGATGTTATTCGCAAAGG-3′ and reverse 5′-GGAGGCTTCCGGGTCAAG-3′; Forward 5′-TGTTCCAACACTGACATATATAGCATCA-3′ and reverse 5′-TGCCAAGTTTCATTATCTGAATCAA-3′; Forward 5′-ACTGAAGCAAAGGATGCATCTG-3′ 3′ and reverse 5′-TTTTCGATTGGCGTCTTAGGA-3′	5′-CCGTCAGACCTCTGGCAGTACCTTCCTC-3′; 5′-ATGCTATAAAATACACAGGAGCCACCCCCA-3′; 5′-CACCACCAGCATCTCCGCCTGC-3′	TaqMan probes labelled with fluorescent dyes CAL Fluor Orange 560, Quasar 670, Fluorescein amidite (FAM), and 5-Carboxytetramethylrhodamine (TAMRA), respectively	NA	[75]
Real-Time	Meat (PND)	Spiked *Listeria monocytogenes*: *ilyA* gene	Forward 5′-GGCTTTCAGCTGGGCATAACCAA-3′ and reverse 5′-GCGGTCAGTGTAAAAAGTGGCACA-3′	NA	Brilliant SYBR Green QPCR Master Mix	1 cfu/g	[76]
Simple	Meat (Breasts, drumsticks, legs)	*Arcobacter* spp.: 16S rRNA	Forward 5′-AGAACGGGTTATAGCTTGCTAT-3′ and reverse 5′-GATACAATACAGGCTAATCTCT-3′	NA	NA	NA	[77]
Real-Time Quantitative	Meat (Breasts, wings, legs)	*Campylobacter jejuni*: *rpoB* gene	Forward 5′-GAGTAAGCTTGGTAAGATTAAAG-3′ and reverse 5′-AAGAAGTTTTAGAGTTTCTCC-3′	NA	FluoCycle SYBR GreenMix	10 cfu/g	[78]
Simple	Meat (PND)	*Arcobacter*, *butzleri:* 16S rRNA, *A. cryaerophilus*, *A. skirrowii*, *A. cibarius*, *gyrA* gene	Forward 5′-AGTTGTTGTGAGGCTCCAC-3′ and reverse 5′-GCAGACACTAATCTATCTCTAAATCA-3′; Forward 5′-TGCTAAAATTGCAGATGTACCA-3′; and reverse 5′- AATTCCTTTTTCAGAAACTGTACG-3′; Forward 5′- GAGACAACTTTTGGAACTATTCTATGA-3′ and reverse 5′-GAAGATAGATTAACTTTTGCTTGTTG-3′; Forward 5′- TGGAAATATTGTTGGTGAAGTTCAG-3′ and reverse 5′- ATCTACATTTACAATACTTACTCCCGAA-3′	NA	NA	NA	[79]
Multiplex	Meat (PND)	Spiked *Salmonella* spp*. invA*, *sdf*, STM4492 genes	Forward 5′-AAA CGT TGA AAA ACT GAG GA-3′ and reverse 5′-TCG TCA TTC CAT TAC CTA CC-3′; Forward 5′-AAA TGT GTT TTA TCT GAT GCA AGA GG-3′ and reverse 5′-GTT CGT TCT TCT GGT ACT TAC GAT GAC-3′; Forward 5′-ACA GCT TGG CCT ACG CGA G-3′ and reverse 5′-AGC AAC CGT TCG GCC TGA C-3′	NA	NA	10^5^ cfu/mL	[80]
Multiplex Real-Time	Meat (Skin)	Spiked *Salmonella* spp.: *invA*, *Campylobacter* spp.: 16S rRNA	Forward 5′-TCGTCATTCCATTACCTACC-3′ and reverse 5′-AAACGTTGAAAAACTGAGGA-3′; Forward 5′-CTGCTTAACACAAGTTGAGTAGG-3′ and reverse 5′-TTCCTTAGGTACCGTCAGAA-3′	5′-TCTGGTTGATTTCCTGATCGCA-3′; 5;′- TGTCATCCTCCACGCGGCGTTGCTGC-3′	Cyanines, Fluorescein amidite and VIC fluorophores	1 and 10^6^ cfu/mL	[81]
Real-Time Loop-mediated isothermal amplification	Meat (PND)	Spiked *Salmonella* spp.: gene62181533	Forward 5′-TGA TACTGT GTC TGC GTC CC-3′ and reverse 5′-CGG AGC GGA TAAACG GAG TT-3′	NA	NA	7 cfu/mL	[82]
Multiplex Real-Time	Meat (Skin)	Spiked *Salmonella* spp.: *invA*, *Campylobacter* spp.: 16S rRNA	Forward 5′-TCGTCATTCCATTACCTACC-3′ and reverse 5′-AAACGTTGAAAAACTGAGGA-3′; Forward 5′-CTGCTTAACACAAGTTGAGTAGG-3′ and reverse 5′-TTCCTTAGGTACCGTCAGAA-3′	5′-TCTGGTTGATTTCCTGATCGCA-3′; 5′-TGTCATCCTCCACGCGGCGTTGCTGC-3′	Labeled with Fluorescein amidite (FAM), Cyanines, and VIC fluorophores	1; 10^2^ cfu/mL	[83]

PND—parts not defined; NA—not applicable.

**Table 5:** Array-based approaches used for the detection of chicken-borne pathogens.

Sample Used	Target Site of Bacterial Pathogen	Probe	Array Matrix	Limit of Detection	Reference
Meat (Breast, wings, thighs)	Spiked *Salmonella* spp.: *fimY*, *Shigella* spp.: *ipaH*, *Listeria monocytogenes*: *prfA*, *Escherichia coli*: *uspA* genes	FY5′-GCCTCAATACAGGAGACAGGTAGCGCC-3′; 5′-ATATCGCTTTGTTGCCAACTGAGCGC-3′; 5′-AAATAAGTAGTGACTCAATGAATAGCCGAG-3′; 5′-AGTTGTAATTATTGCCTGAGAAATGATAC-3′, IH5′-GGGAGTGACAGCAAATGACCTCCGC-3′; 5′-CGGCACTGGTTCTCCCTCTGGGGACCA-3′; 5′-TGTGGATGAGATAGAAGTCTACCTGG-3′; 5′-AGAATGAGTACTCTCAGAGGGTGGCTGAC-3′; 5′-AGAAACTTCAGCTCTCCACTGCCGTGA-3′, PA5′-ACGGGAAGCTTGGCTCTATTTTGCGG-3′; 5′-AGCTTACAAGTATTAGCGAGAACGGGACCA-3′; 5′-ACAAAGGTGCTTTCGTTATAATGTCTGGCT-3′; 5′-AATTTAGAAGTCATTAGCGAACAGGCT-3′; 5′-CATACAGCCTAGCTAAATTTAATGAT-3′; 5′-AAACATCGGTTGGCTATTATAAGTTTAG-3′, UA5′-AAGAGACACATCATGCGCTGACCGAGCT-3; 5′-GGTAGAGAAAGCAGTCTCTATGGCTCGCCC-3′; 5′-ACCGTTCACGTTGATATGCTGATTGTTCCG-3′; 5′-TTGTTTATCTAACGAGTAAGCAAG-3′; 5′-AAGGTAAGGATGGTCTTAACACTGAAT-3′; 5′-GGTGACGTAACGGCACAAGAAACGCTAGCT-3′	Nylon membrane	10^3^ cfu/mL	[86]
Meat (Breast, wings, thighs)	Spiked *Salmonella* serotype *enteritidis*: *fimY*, *Listeria monocytogenes*: *prfA*, *Shigella boydii*: *ipaH* genes	FY5′-GCCTCAATACAGGAGACAGGTAGCGCC-3′; 5′-ATATCGCTTTGTTGCCAACTGAGCGC-3′; 5′-AAATAAGTAGTGACTCAATGAATAGCCGAG-3′; 5′-AGTTGTAATTATTGCCTGAGAAATGATAC-3′, PA5′-ACGGGAAGCTTGGCTCTATTTTGCGG-3′; 5′-AGCTTACAAGTATTAGCGAGAACGGGACCA-3′; 5′-ACAAAGGTGCTTTCGTTATAATGTCTGGCT-3′; 5′-AATTTAGAAGTCATTAGCGAACAGGCT-3′; 5′-AAACATCGGTTGGCTATTATAAGTTTAG-3′, IH5′-GGGAGTGACAGCAAATGACCTCCGC-3′; 5′-CGGCACTGGTTCTCCCTCTGGGGACCA-3′; 5′-TGTGGATGAGATAGAAGTCTACCTGG-3′; 5′-AGAATGAGTACTCTCAGAGGGTGGCTGAC-3′; 5′-AGAAACTTCAGCTCTCCACTGCCGTGA-3′	Nylon membrane	10^4^–10^6^ cfu/mL	[87]
Meat (PND)	Spiked *Salmonella enteritidis: sdf*, *Salmonella typhimurium*: *STM4497* gene, *Campylobacter jejuni*: *hipO*, *Campylobacter coli*: *ceuE* gene	Btn-TG-T10-AATCAGCCTGTTGTCTGCTCACCATTC-3′; Btn-TG-T10-AGATCATCGTCGACATGCTCAC-3′, Btn-TG-T10-CATTGCGAGATACTATGCTTTG-3′, Btn-TG-T10-CTGTAAGTATTTTGGCAAGTTT-3′	DVD chips	0.2 pg genomic DNA	[88]

PND—parts not defined.

**Table 6:** ELISA-based approaches used for the detection of chicken-borne pathogens.

Type of ELISA	Sample Used	Target Site of Bacterial Pathogen	Sensitivity	Limit of Detection	Reference
Indirect	Meat (Thighs, legs)	Outer membrane protein of *Salmonella enterica* serovar Enteritidis	94%	NA	[91]
Sandwich	Spiked wings	*Salmonella* spp.	75%	1.6 × 10^3^ cfu/mL	[92]
Sandwich	Meat (PND)	*Campylobacter* spp.	ND	NA	[93]
Sandwich	Spiked meat (PND) and naturally contaminated	*Salmonella* spp.	ND	5 × 10^3^ cfu/mL	[94]

PND—parts not defined; ND—not defined; NA—not applicable.

**Table 7:** Aptamers based approaches used for the detection of chicken-borne pathogens.

Sample Used	Target Site of Bacterial Pathogen	Aptamer Name and Sequence	Detection Format	Limit of Detection	Reference
Spiked meat	*Listeria monocytogenes*: *InlA* gene	A8, 5′-ATC CAT GGG GCG GAGATG AGG GGG AGG AGG GCG GGT ACC CGG TTGAT-3′, A610.2, 5′- GGT TACTGA AGC ATA TGT CCG GGG GAT TGC CAA GCCTTC CC-3′	Sandwich ELISA	10^3^ cfu/mL	[104]
Spiked meat	Whole-cell of *Salmonella enterica* serovar Typhimurium	ND, 5′-TATGGCGGCGTCACCCGACGGGGACTTGACATTATGACAG-3′	Electrochemical	10^1^ cfu/mL	[105]
Spiked meat (Breast)	Whole cell of *Salmonella typhimurium*	ND, 5′-NH_2_-ATAGGAGTCACGACGACCAGAAAGTAATGCCCGGTAGTTATTCAAAGATGAGTAGGAAAAGATATGTGCGTCTACCTCTTGACTAAT-3′	FRET	35 cfu/mL	[106]
Spiked cooked meat	Whole-cell of *Streptococcus pyogenes*	S2, 5′-GTTCGGGGTCGGGGTGAGTGGGGCCTAGGAGTGGGGGCGC-3′, S8, 5′-ATGGGGGGCGGGGAGGTGGGTACAGGGTCGGGGATGGCAG-3′, S10, 5′-CGGGCGGGGCGTGGGGTGTTGGAGTGGAGGGCGGGGCGGC-3′, S12, 5′-GCGGGCGGGGGGAGGGCGGCCGTGGGCTGCGAGTGGGAGG-3′, S15, 5′-CAGGGTGCGGGAGGGCCAAAGGGGGGAGGGCCCGGGGGGA-3′	FRET	70 cfu/mL	[107]
Spiked chicken	*E. coli* O157: H7	F1N, 5′-ATAGGAGTCACGACGACCAGAA, R1N, ATTAGTCAAGAGGTAGACGCACATA, Bio Rev, 5Biosg/ATTAGTCAAGAGGTAGACGCACATA	Quantum dots	10^2^ cfu/mL	[108]

ND—not defined; FRET—fluorescence resonance energy transfer.

**Table 8:** Biosensor-based approaches used for the detection of chicken-borne pathogens.

Biosensor Type	Sensing Platform	Chicken Matrix	Pathogens/Toxins	Limit of Detection	Analysis Time	Reference
Phage magnetoelastic	Gold electrode	Boneless and skinless breast fillets	Spiked *Salmonella enterica* serovar Typhimurium	7.86 × 10^3^ cfu/mm^2^	2–10 min	[116]
Multiplex fiber optic	Polystyrene waveguides	Breast	Spiked *Listeria monocytogenes*, *Escherichia coli* O157:H7, *Salmonella enterica*	10^3^ cfu/mL	<24 h	[117]
Fiber optic	Polystyrene waveguides	Breast	Spiked *Salmonella enteritidis*	10^2^ cfu/mL	<8 h	[118]
Colorimetric	C2 reverse-phase silica gel plates with sensitive dyes	Breast fillets	Spiked *Pseudomonas gessardii*, *Pseudomonas psychrophila*, *Pseudomonas fragi*, *Pseudomonas fluorescens*	NA	ND	[119]
Dithiobis-succinimidyl propionate-modifiedimmunosensor	Gold electrode	Skin	Spiked *Listeria monocytogene*	10^3^ cfu/25 g	45 min	[120]
Amperometric	Screen-printed electrode	NS	*Salmonella pullorum*	100 cfu/mL	1.5–2 h	[121]
Optical scattering	SELA plates	Breast	Spiked *Listeria monocytogenes*, *Escherichia coli* O157: H7, *Salmonella enteritidis*	ND	29–40 h	[122]
Colorimetric	Aptamer	NS	*Campylobacter coli*, *Campylobacter jejuni*	7.2 × 10^5^ cfu/mL	30 min	[123]

SELA—*Salmonella*, *Escherichia* and *Listeria* agar; NS—not specified; NA—not applicable; ND—not defined.

**Table 9:** Microbial inhibition-based approaches used for the detection of antibiotics residue in chicken meat.

Meat Sample	Method Type	Types of Antibiotics Residue	Microbial Test Strains	Reference
Muscles, kidney, liver, gizzard	Three-Plate test	Tetracycline, β-lactams, sulphonamides, aminoglycosides	*Bacillus subtilis*	[143]
Spiked liver, kidney, breast, thigh muscle, skin	ND	Enrofloxacin, ciprofloxacin, oxytetracycline	*Geobacillus stearothermophilus*	[144]
Breast, liver, thigh tissue	Four-Plate test	Tetracycline	*Bacillus subtilis*	[145]
Breast	Four-Plate test	Tetracycline, β-lactams, sulphonamides, aminoglycosides	*Bacillus subtilis*, *Micrococcus luteus*	[146]
Fillet	ND	Oxytetracycline, enrofloxacin	*Bacillus subtilis*	[147]
Breast, thighs	Four-Plate test	Tetracycline, β-lactams, sulphonamides, aminoglycosides, macrolides, quinolones	*Bacillus subtlis* spore, *Micrococcus luteus*, *Escherichia coli*	[148]
Liver, kidney, muscle	Four-Plate test	Chloramphenicol	*Bacillus subtilis*, *Staphylococcus aureus*	[149]

ND—not defined.

**Table 10:** ELISA-based approaches used for the detection of antibiotic residue in chicken meat.

Type of ELISA	Sample Used	Target Antibiotic	Limit of Detection	Reference
Competitive	Breast, liver, thigh tissue	Tetracycline	0.05 µg/Kg	[145]
Competitive	Liver, kidney	Ciprofloxacin, streptomycin, sulphanilamide, tetracycline	10 ppb	[150]
ND	Breast	Enrofl oxacin, ciprofloxacin, streptomycin, chloramphenicol	ND	[153]
Competitive	Breast	Tetracycline, streptomycin, chloramphenicol, sulfamethazine	ND	[151]
ND	Breast	Tetracycline	ND	[154]
Indirect competitive	Spiked muscles	Chloramphenicol	6 ng/L	[152]
ND	Muscles, liver, kidney	Gentamicin	0.05 µg/Kg	[155]
Competitive	Breast	Quinolone	0.05 µg/Kg	[28]
Competitive	Breast	Quinolone	0.05 µg/Kg	[156]

ND—not defined.

**Table 11:** TLC-based approaches used for the detection of antibiotic residue in chicken/meat.

Sample Used	Stationary Phase	Mobile Phase	Target Antibiotic	Reference
Breast, thigh muscle, liver	Silica	Acetone and Methanol: 1:1	Ciprofloxacin, enrofloxacin, oxytetracycline, doxycycline, amoxicillin	[159]
Liver	Silica	Acetone and Methanol: 1:1	ND	[160]
Liver, kidney	Silica	Acetone and Methanol: 1:1	Sulphanilamide, streptomycin, ciprofloxacin, tetracycline	[150]
Oral administration of chicken blood	Silica	Acetone and Methanol: 1:1	Ciprofloxacin	[161]
Spiked muscles	Silica	Chloroform and n-Butanol: 90:10	Sulfadiazine, sulfadoxine, sulfamethazine, sulfathiazole, sulfaquinoxaline	[162]
MPND	Silica	Acetone and Methanol: 1:1	Doxycycline, oxytetracycline	[163]
Breast, thigh muscle, liver, kidney	Silica	Acetone and Methanol: 1:1	Doxycycline	[164]

MPND—meat portion not defined; ND—not defined.

**Table 12:** HPLC-based approaches used for the detection of antibiotics residue in chicken meat.

Sample Used	Types of Antibiotic	Method Used	Chromatography Conditions Used				Limit of Detection	Reference
			Model	Column	Solvent	Flow Rate		
Breast, liver, thigh tissue	Tetracycline	HPLC-UV	KNAUER liquid chromatography system, Berlin, Germany	Eurospher RP-C_18_ column (250 × 4.6 mm i.d.). A guard column (Eurospher 100-5 C_18_) was used to protect the analytical column	Mobile phase was a gradient elution using MeOH; acetonitrile; 0.03 M oxalic acid buffer pH 2.5; water	0.9 mL/min	25 µg/Kg	[145]
Spiked breast, thigh, liver, kidney	Oxytetracycline, tetracycline	HPLC-UV	HPLC (Shimadzu Corporation, Tokyo, Japan)	Inertsil ODS-3 column	Mobile phase consisting of methanol:acetonitrile: 0.01 M oxalic acid dihydrate (5:18:77 *v*/*v*/*v*)	1 mL/min	50 ng/mL	[168]
Spiked meat, liver	Oxytetracycline, tetracycline, chlortetracycline, doxycycline	HPLC-DAD	The HPLC system of a HP 1100 chromatograph (Agilent Technologies, Palo Alto, CA, USA)	The analytical column was reversed-phase (Nuclosil 100 C_18_, 25 cm × 4.6 mm I.D., 5 µm, Germany)	The mixture of acetonitrile/0.03 M oxalic acid (40:60, *v*/*v*); The mixture of methanol/acetonitrile/0.03 M oxalic acid (10:30:60, *v*/*v*/*v*); The mixture of methanol/acetonitrile/0.03 M oxalic acid (20:20:60, *v*/*v*/*v*)	1. 1 mL/min; 2. 1 mL/min; 3. 1 mL/min; 4. NS; 5. NS	4.4, 5, 13 and 10 ng/g	[169]
Spiked muscle	Marbofloxacin, ciprofloxacin, norfloxacin, lomefloxacin, danofloxacin, enrofloxacin, sarafloxacin, difloxacin, oxolinic acid, flumequine	HPLC-FAD	HPLC system (Waters, Milford, MA, USA)	The reverse phase analytical column was a Symmetry C_18_ (250 mm × 4.5 mm i.d., 5 µ m) from Waters	Mobile phase consisted of aqueous formic acid solution (0.02%, pH 2.8) and acetonitrile	1.0 mL/min	0.3–1.0 ng/g	[170]
Spiked muscle	Ofloxacin, norfloxacin, ciprofloxacin, enrofloxacin, oxytetracycline, tetracycline, chlortetracycline, doxycycline, sulfadiazine, sulfamethazine, sulfadimethoxydiazine, sulfamonomethoxine, sulfamethoxazole, sulfaquinoxaline	UPLC-MS-MS	UPLC system (Waters, Milford, MA, USA)	UPLC BEH C_18_ column(50 mm 9 2.1 mm i.d., 1.7 lm) from Waters	Mobile phase, consisting of methanol (solvent A) and 0.01% formic acid in water (solvent B)	0.3 mL/min	0.3 µg/Kg	[171]
Spiked muscle, liver, kidney	Amoxicillin, amoxicillin metabolites, ampicillin	UPLC-MS-MS	UPLC system (Waters, Milford, MA, USA)	UPLC HSS T3 column (100 × 2.1 mm, internal diameter (i.d.) 1.8 μm)	A (0.15% formic acid) and B (acetonitrile)	0.5 mL/min	0.01–1.36 µg/Kg	[172]

NS—not specified.

**Table 13:** Biosensor-based approaches used for the detection of antibiotics residue in chicken meat.

Biosensor Type	Sensing Platform	Chicken Matrix	Antibiotic	Limit of Detection	Analysis Time	Reference
Bioluminescent biosensor	Bacteria *E. coli* K12	Spiked breast fillet	Tetracycline	100 ng/g	4 h	[173]
Electrochemical	Gold and platinum nanowire	Spiked breast	Penicillin and tetracycline	41.2 μA μM^−1^ cm^−2^ and 26.4 μA μM^−1^ cm^−2^	ND	[175]
Electrochemical	Glassy carbon electrode	PND	Tetracycline	0.10 µM	ND	[176]
Electrochemical	Pencil graphite electrode	Spiked PND	Sulfadimethoxine	3.7 × 10^−16^ M	ND	[177]
Amperometric	Glassy carbon electrode	PND	Chloramphenicol	45 pg/mL	ND	[178]
Surface plasmon resonance	NS	Spiked muscle	Chloramphenicol and chloramphenicol glucuronide	ND	ND	[179]
Bioluminescent biosensor bacteria	Bacteria *E. coli*	Spiked muscle	Tetracycline	ND	ND	[175]
Surface plasmon resonance	NS	Spiked breast	Norfloxacin, sarafloxacin, difloxacin, ciprofloxacin, enrofloxacin, flumequine, danofloxacin, marbofloxacin, pefloxacin, enoxacin, lomefloxacin, ofloxacin, oxolinic acid	ND	ND	[180]

NS—not specified; PND—portion not defined; ND—not defined.
